# Supplementary material for: Nonlinear effects of noise on outbreaks of mosquito-borne diseases
Source: PLoS Comput Biol. 2026 Apr 13;22(4):e1013466. doi: 10.1371/journal.pcbi.1013466 (PMC13095129; doi:10.1371/journal.pcbi.1013466)

S1 Text: Supplementary figures for Nonlinear effects of noise on outbreaks of mosquito-borne diseases

Kyle J.-M. Dahlin[1,2,*], Karin Ebey[1,3], John E. Vinson[1,4], John M. Drake[1,5]

1 Odum School of Ecology and Center for the Ecology of Infectious Diseases, University of Georgia, Athens, Georgia, United States of America
2 Department of Mathematics, Virginia Polytechnic Institute and State University, Blacksburg, Virginia, United States of America
3 Department of Biology, Eckerd College, St. Petersburg, Florida, United States of America
4 Center for Wildlife Sustainability Research, Southern Illinois University, Carbondale, Illinois, United States of America
5 Pandemic Sciences Institute, University of Oxford, Oxford, United Kingdom

* Corresponding author: kydahlin@gmail.com

Outbreak duration vs. intensity

Measurements of the outbreak duration and intensity from 10,000 simulation trajectories of the full model. Points are colored by the number of their nearest neighbors, indicating the degree of clustering around each point. Each panel is a combination of $\mathcal{R}_0$ (rows) and environmental noise strength (σ; columns). The red diamonds indicate the mean values of duration and intensity.

Fig A. Comparing outbreak duration and intensity.

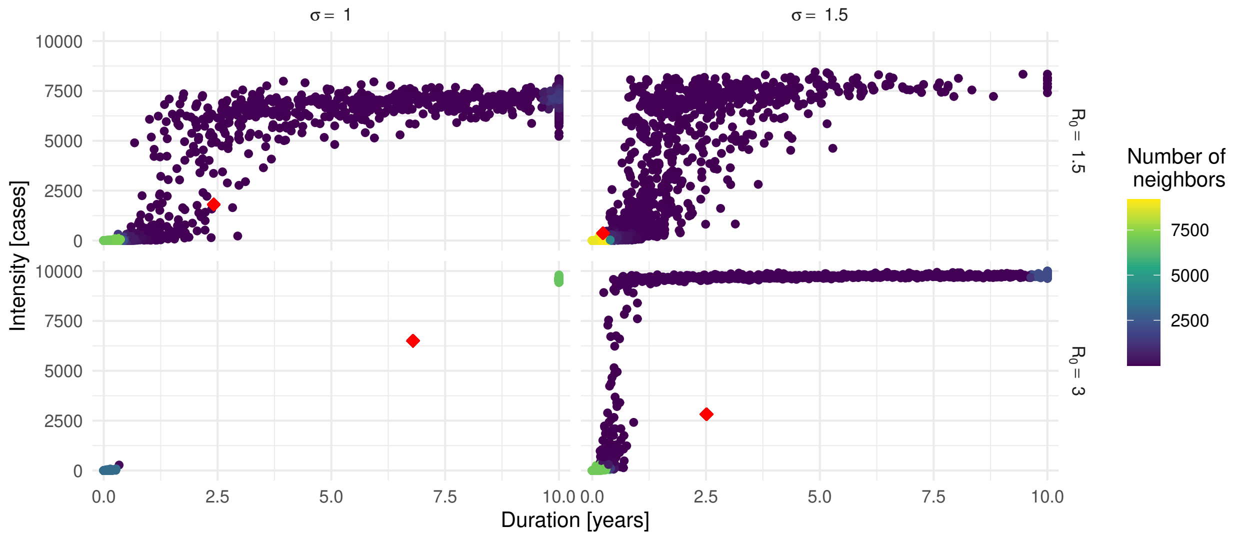

Sensitivity analysis

In the following figures, the solid black line indicates the mean across 1,000 simulations. The bars indicate the range from the bottom quartile to the upper quartile. The bars are colored to aid in differentiating the subplots. The columns indicate the value of τ_{HV} used in the simulations. These values correspond to the values of $\mathcal{R}_0$ in the manuscript. The rows are in increasing order of the chosen variable (top to bottom).

The figures are organized as follows: outbreak probability (Figures S1-S4), outbreak intensity (Figures S5-S8), and outbreak duration (Figures S9-S12).

Outbreak probability figures

Fig B. Outbreak probability varying biting rate, b

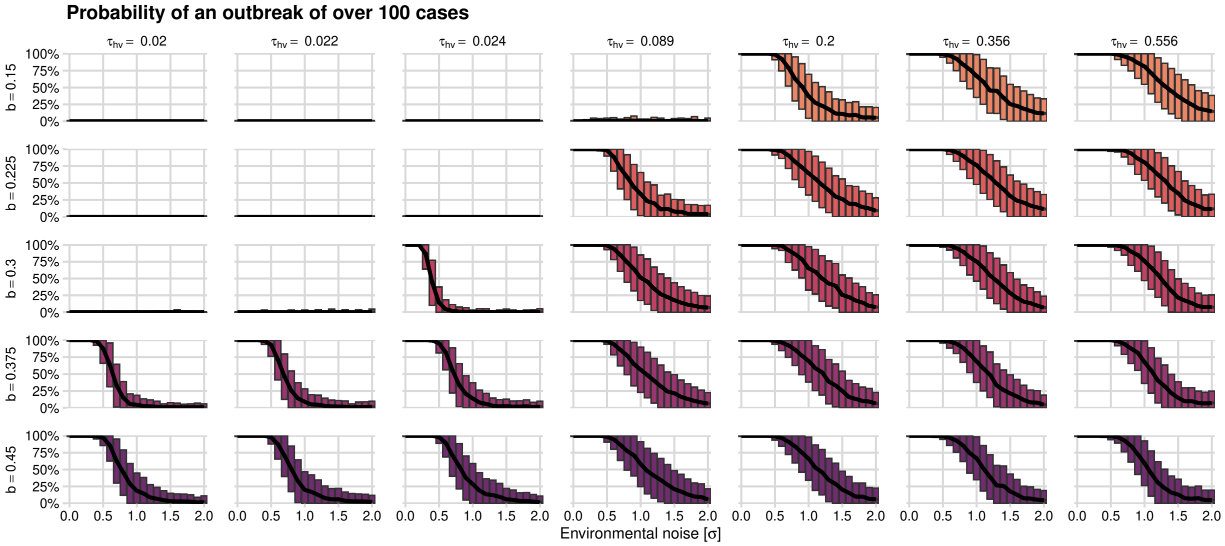

Fig C. Outbreak probability varying host population size, N_H.

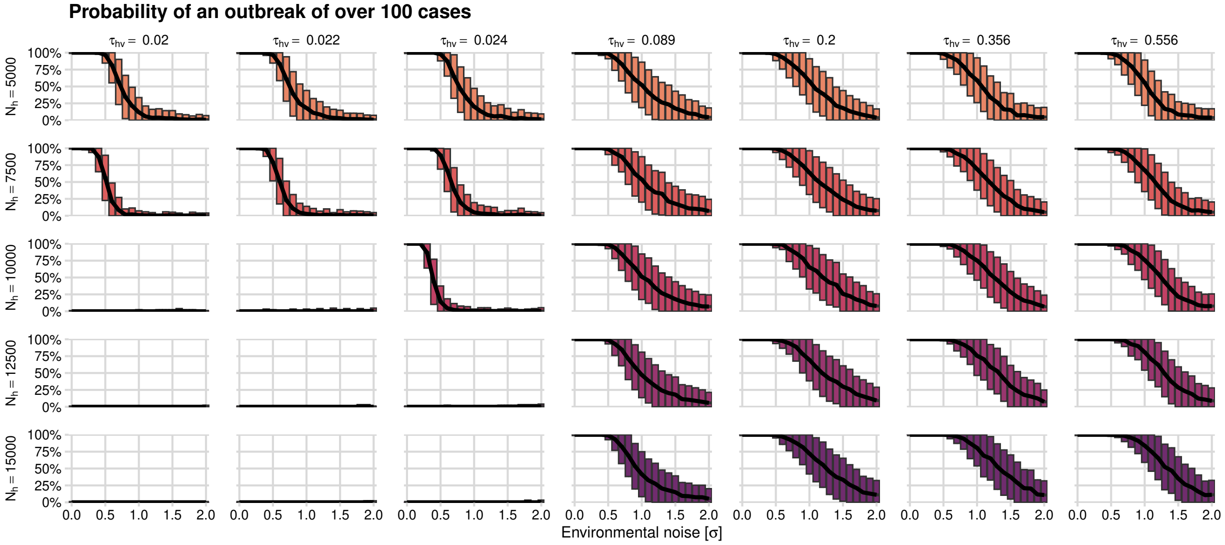

Fig D. Outbreak probability varying vector population size, N_V.

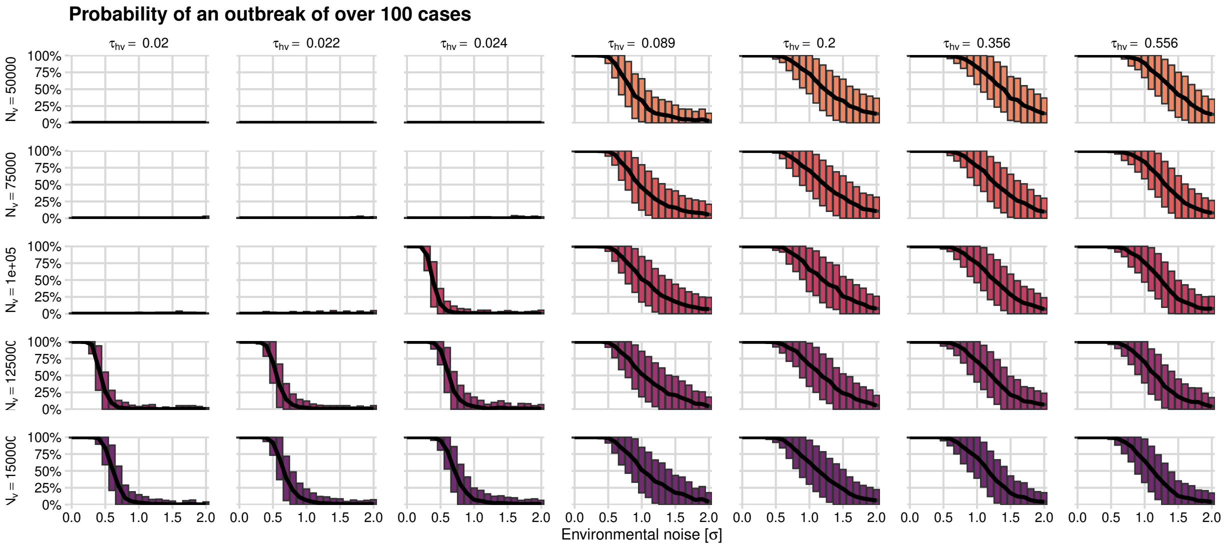

Fig E. Outbreak probability varying vector-to-host transmission probability, τ_{VH}.

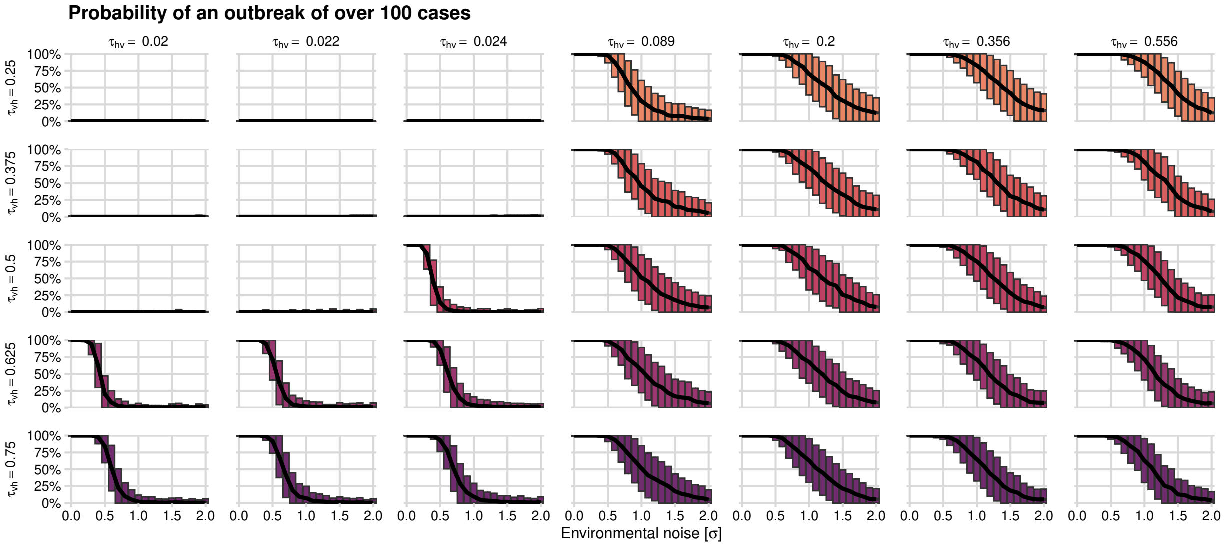

Outbreak intensity figures

Fig F. Outbreak intensity varying biting rate, b

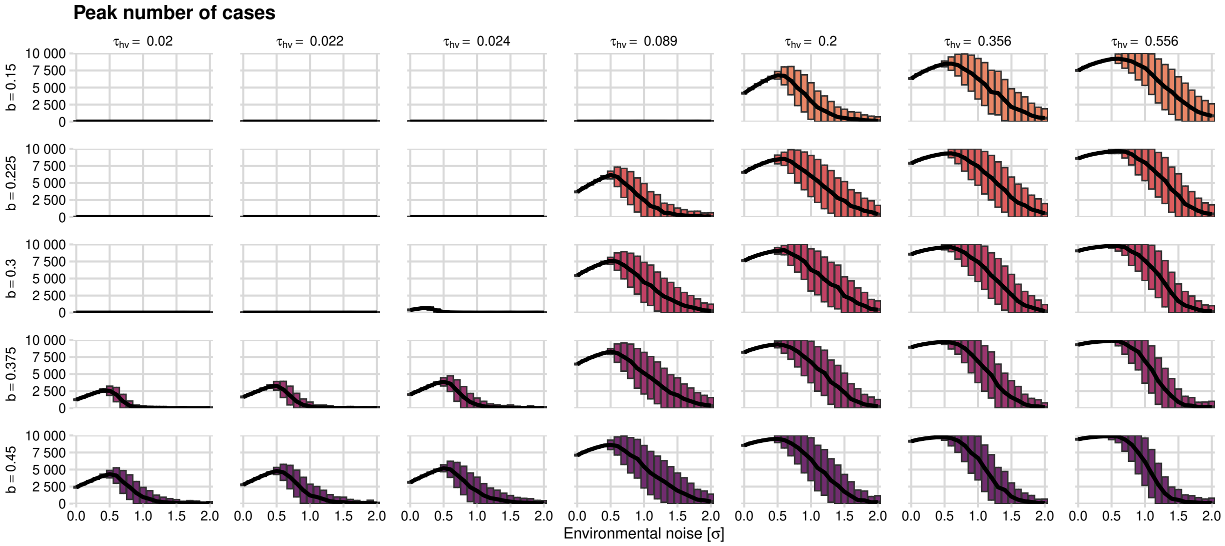

Fig G. Outbreak intensity varying host population size, N_H.

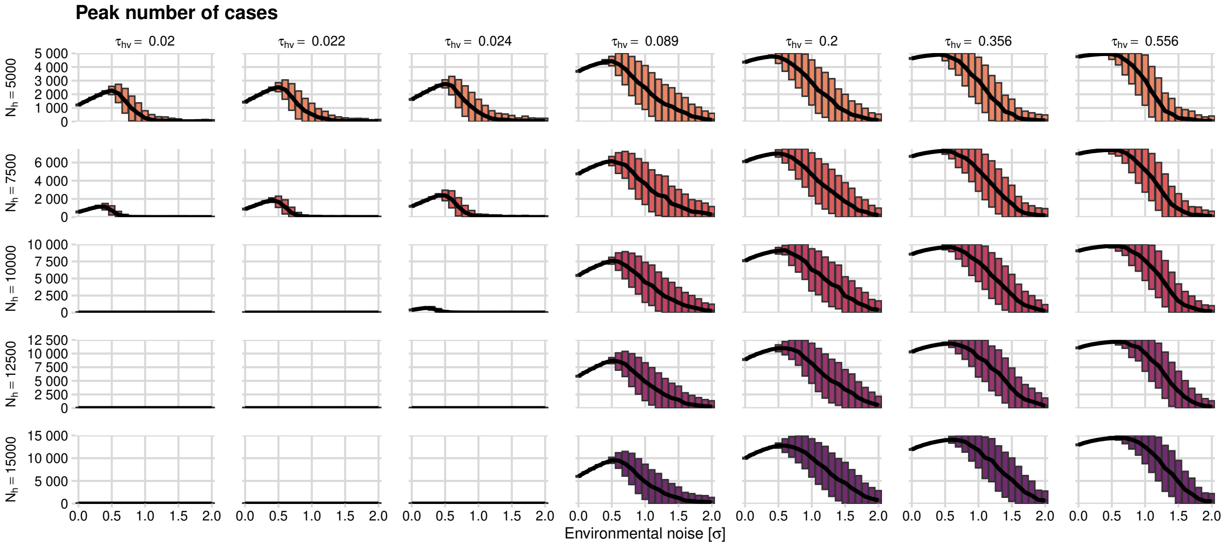

Fig H. Outbreak intensity varying vector population size, N_V.

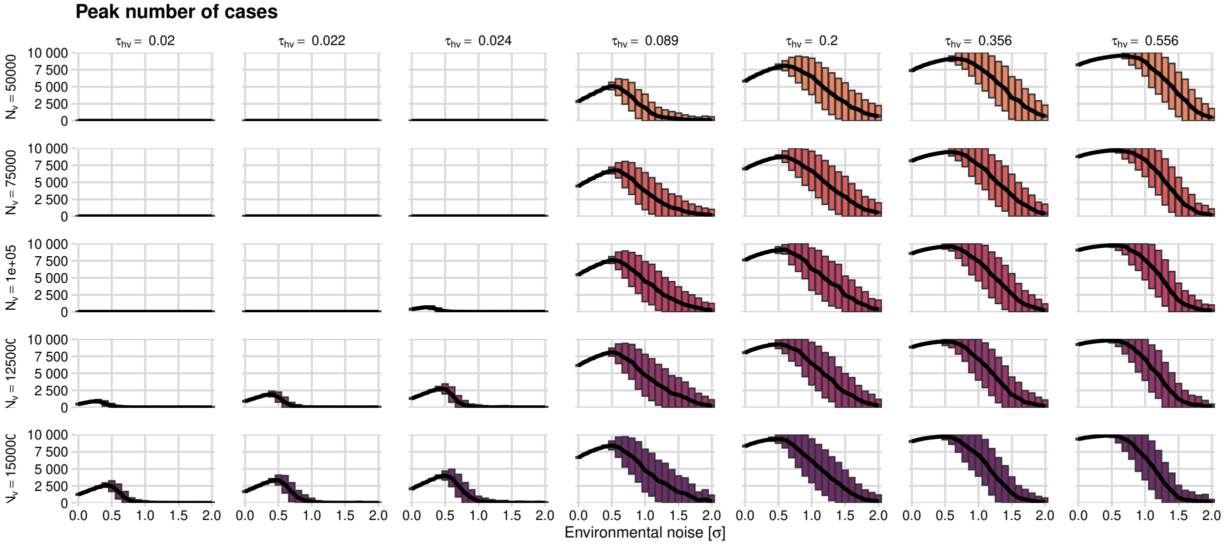

Fig I. Outbreak intensity varying vector-to-host transmission probability, τ_{VH}.

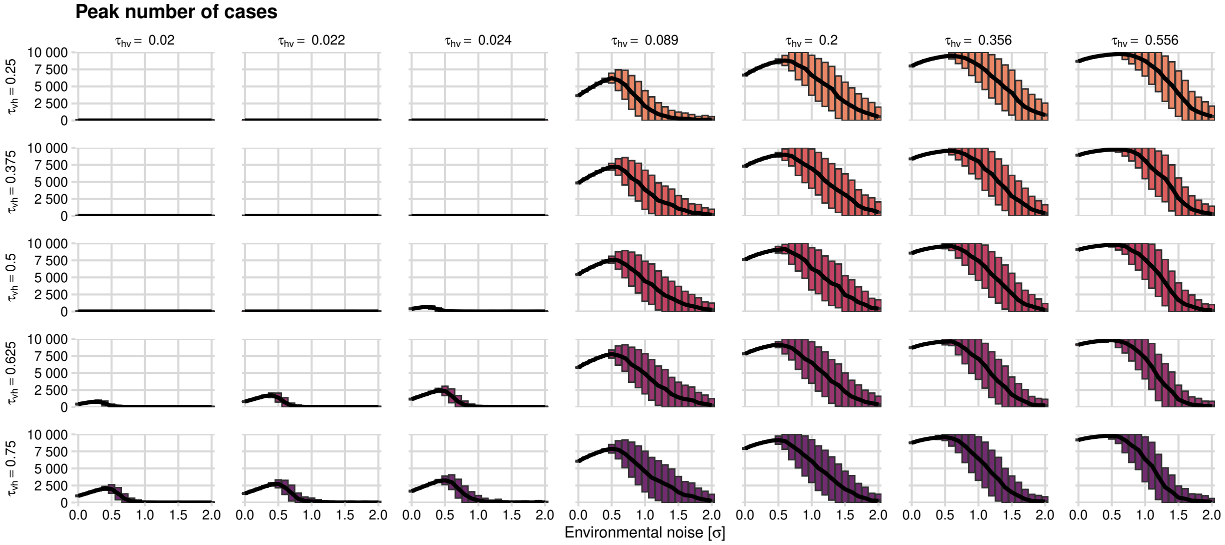

Outbreak duration figures

Fig J. Outbreak duration varying biting rate, b

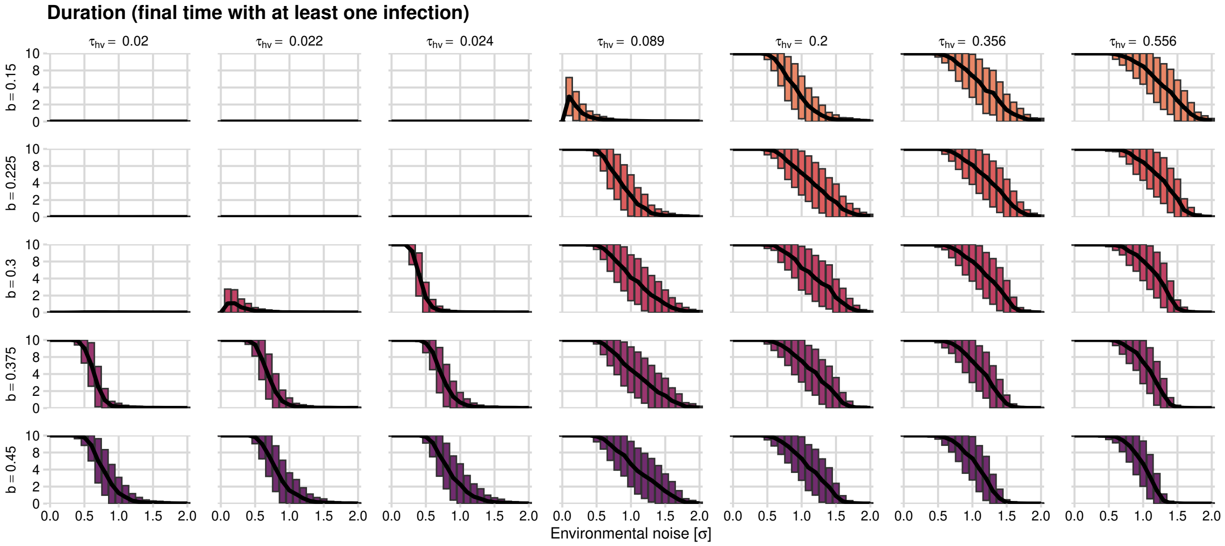

Fig K. Outbreak duration varying host population size, N_H.

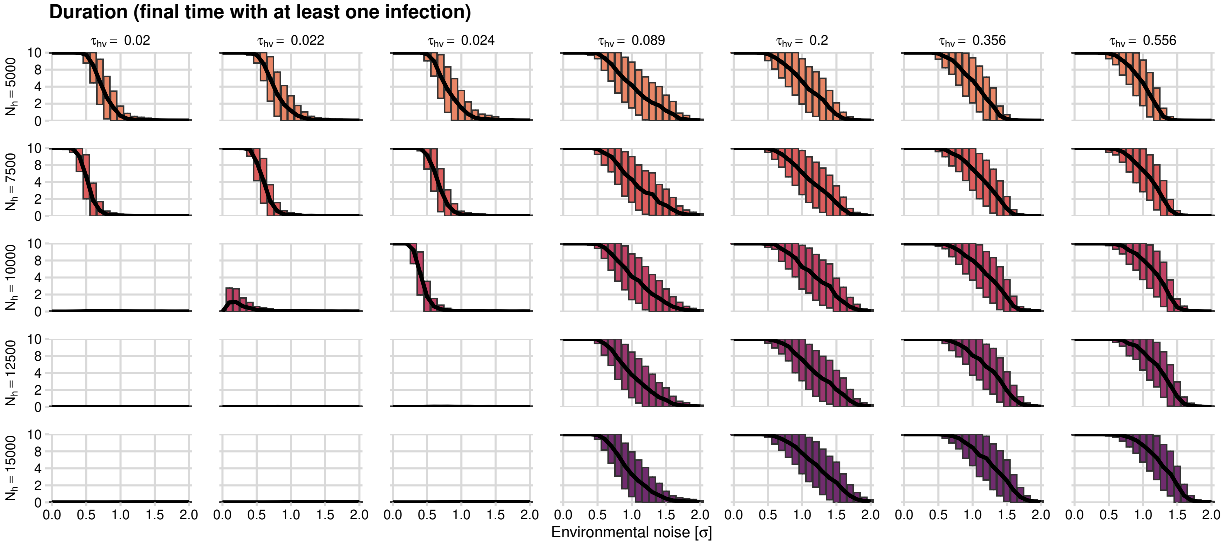

Fig L. Outbreak duration varying vector population size, N_V.

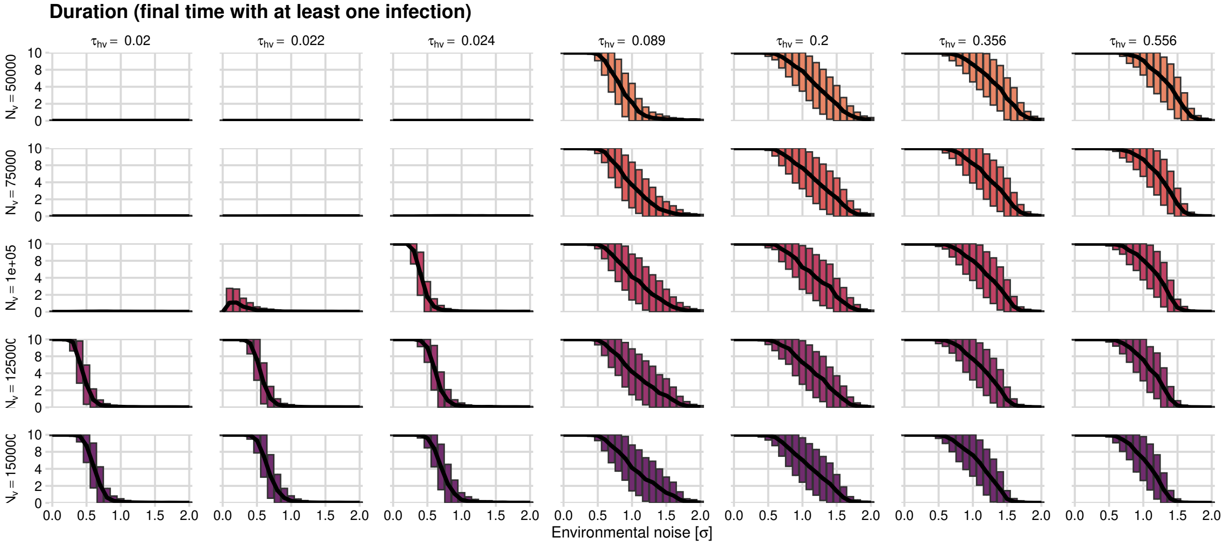

Fig M. Outbreak duration varying vector-to-host transmission probability, τ_{VH}.

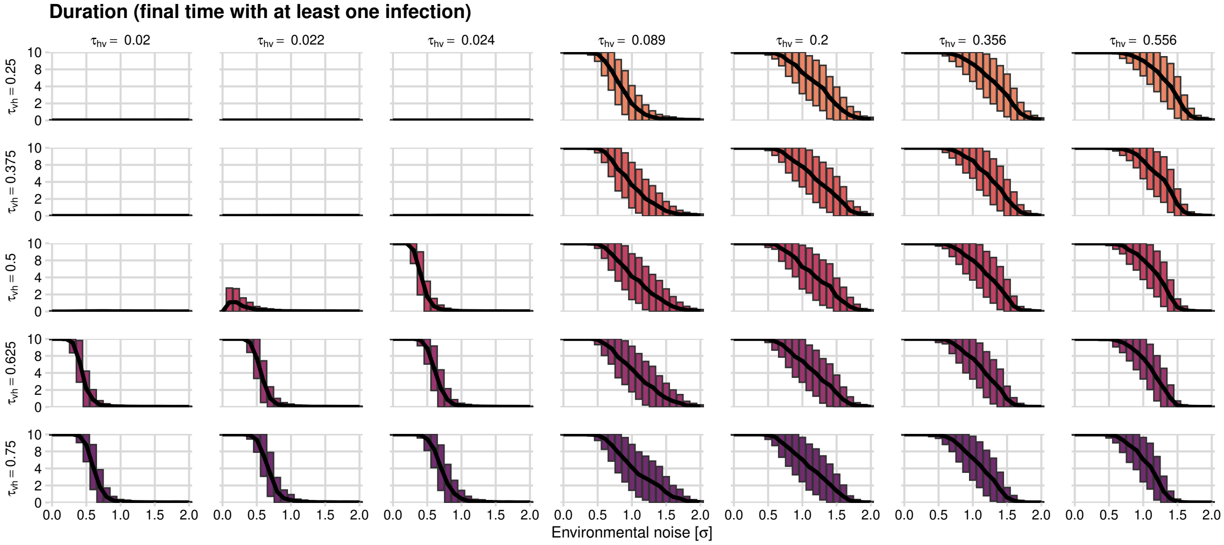

Supplement: S1 Text — Supplementary captions from Fig A-Fig M. (PDF) [file pcbi.1013466.s001.pdf]
